# Evaluation of the effect of the elective blastocyst-stage embryo transfer and freezing strategy on the abandonment of frozen embryos under the Taiwan National Assisted Reproduction Act

**DOI:** 10.1007/s10815-020-01699-5

**Published:** 2020-01-28

**Authors:** Kuo-Chung Lan, Ya-Jung Tseng, Yi-Ru Su, Tzu-Yu Lin, Yi-Chi Lin

**Affiliations:** 1 Department of Obstetrics and Gynecology, Kaohsiung Chang Gung Memorial Hospital and Chang Gung University College of Medicine, 123 Ta-Pei Road, Niao-Sung District, Kaohsiung City, Taiwan; 2 Center for Menopause and Reproductive Medicine Research, Kaohsiung Chang Gung Memorial Hospital and Chang Gung University College of Medicine, Kaohsiung, Taiwan

**Keywords:** Abandoned embryo, Assisted reproduction act, Blastocyst transfer, Frozen embryos, Good prognosis

## Abstract

**Purpose:**

To evaluate the relationship between elective blastocyst transfer, freezing strategy, and the abandonment of frozen embryos with a storage time limit of 10 years as specified in the National Assisted Reproduction Act of Taiwan.

**Methods:**

This two-phase retrospective cohort study was conducted at a single tertiary center, Kaohsiung Chang Gung Memorial Hospital (KCGMH), in 2019. Participants were selected from a data registry containing 4167 fresh IVF cycles, including phase 1 cycles from 1999 to 2009 and phase 2 cycles from 2010 to 2014, at KCGMH.

**Results:**

In phase 1, embryo abandonment was associated with the production of more mature oocytes and embryos, the freezing of more embryos, young female age, blastocyst transfer, and positive pregnancy results. After adjustment for confounding factors, only positive pregnancy results (adjusted odds ratio [aOR] 4.38, 95% confidence interval [CI] 3.17, 6.04), the freezing of ≥ 2 embryos (aOR 3.68, 95% CI 3.10, 4.38), the production of ≥ 6 embryos (aOR 1.68, 95% CI 1.03, 2.73), and the use blastocyst transfer (aOR 2.46, 95% CI 1.64, 3.69) remained significantly associated with embryo abandonment. The factors associated with embryo abandonment or possible abandonment were similar in phase 2.

**Conclusion:**

For elective blastocyst stage transfer and a freezing strategy performed according to the Taiwan National Assisted Reproduction Act, a young female age ≤ 35 with positive pregnancy status due to the original IVF treatment, the production of ≥ 6 embryos, and the cryopreservation of ≥ 2 blastocysts may increase the likelihood of abandoning embryos in the future.

## Introduction

The individualization of treatment for in vitro fertilization (IVF) may improve patient compliance, increase the probability of pregnancy, and reduce iatrogenic and avoidable risks, but it can be difficult to implement as expected in clinical practice [1]. Although live birth is the most common clinical outcome following IVF, clinicians and researchers commonly use the number of eggs retrieved following ovarian stimulation as a surrogate outcome measure [2]. Based on studies that included frozen embryo transfer cycles, the cumulative live birth rate significantly increases with the number of oocytes retrieved [3–6]. Indeed, ovarian stimulation increases the likelihood of pregnancy, and the availability of frozen surplus embryos increases the probability of an ultimately successful pregnancy. However, few studies have examined the status of abandoned embryos that result from the production of many oocytes. Frozen storage of supernumerary IVF embryos provides benefits to patients but has led to new concerns [7], such as ethical [8], legal [9], and social issues [10], and the need for counseling for IVF workers who may be required to discard unwanted embryos [11, 12].

Thus, the issue of how to deal with unclaimed excess embryos, for which decisions have not been made or that have been abandoned, continues to be of great concern to clinics. A recent study uniquely provides insight into global embryo disposal practices and trends. The results highlight the divergence between reported practices, with embryologists acknowledging the need for a universally accepted protocol for implementation [13]. National professional organizations have addressed this important issue [14]. If a fertility clinic or storage facility does not have clear directions about what to do with abandoned embryos, if payment for embryo storage ceases, or if the couple who provided the embryos cannot be contacted, the clinic faces practical challenges [9]. Because research in a number of countries has shown at this point that many people with cryopreserved embryos in storage have trouble with decision-making, legal time limits in jurisdictions on storage serve to address this issue, leaving people the option to “abandon” their embryos knowing that they will be disposed of when the time limit elapses [9].

Some countries have legislation regarding the maximum storage period for abandoned human embryos. Although storage limits vary among countries and among states within some countries, most countries stipulate that embryos can remain in storage for up to 5 years [15], although some jurisdictions have a 10-year limit, such as the UK (2009), New Zealand (2010), and certain parts of Australia [16]. The Taiwan National Assisted Reproduction Act, implemented in 2007 and revised in 2018, aimed to improve the development of artificial reproduction; to protect the rights and interests of infertile couples, IVF-born children, and donors; and to maintain the ethical standards and health of people undergoing assisted reproduction in Taiwan. This act says that IVF/ICSI embryos should be destroyed if the marriage of a couple is invalid, the couple divorces, or one party dies; if the embryo was frozen for more than 10 years; or if the couple no longer wants to attempt artificial reproduction. The institution is also not permitted to donate surplus embryos to another couple.

The benefits of blastocyst transfer (BT) are that it facilitates the selection of high-quality embryos, it leads to a high implantation rate, and it lowers the risk of multiple pregnancies. Since 1999, our program has routinely offered elective BT to patients with three or more 8-cell embryos on day-3 [17]. We followed the principles and TSRM (Taiwanese Society for Reproductive Medicine**)** guidelines of recommended limits of the number of embryos to transfer: age < 35, number 1–2; age 35–37, number ≤ 2; age 38–40, number ≤ 3; and age 41, number≤ 4. However, for blastocyst transfer, the number of transfers should be decreased by at least one according the patient’s age and blastocyst grading category. The transfer of a limited number of selected blastocysts is more efficient in achieving successful pregnancy. However, when designing an IVF treatment plan, it is difficult to simultaneously achieve the best cumulative pregnancy rate and to reduce the occurrence of multiple pregnancies and the production of embryos that will ultimately be discarded.

Because of the recent emphasis on cumulative live birth rates in the literature, our study evaluated the use of elective BT and embryo freezing strategies with abandonment of frozen embryos under the Taiwan National Assisted Reproduction Act. “Abandoned embryos” in the USA were redefined by the ASRM to include intentional as well as unintentional abandonment. Thus, the term “abandoned embryos” has been used to describe embryos: (i) in storage for an extended period of time; (ii) where there are no clear written instructions from the gamete and embryo providers about what to do with these embryos if they are not to be thawed for the reproductive use of the individuals or couples for whom they were created; and (iii), for any number of reasons, these individuals or couples cannot be contacted to provide clear written instructions; or (iv) where they are willfully “abandoned.” [9]

Our study tried to identify the relationships between the use of embryos and potential “abandonment” and to evaluate whether elective BT is a useful intervention. Additionally, this study evaluated whether the storage time limit of the National Assisted Reproduction Act of Taiwan has had an effect on decision making related to the disposition of embryos.

## Material and methods

Participants were selected from a data registry of 4167 fresh IVF cycles from January 1, 1999 to December 31, 2014, at Kaohsiung Chang Gung Memorial Hospital (KCGMH). This was a two-phase, retrospective, single-center cohort study. This study was conducted in 2019 because the Taiwan Artificial Reproduction Act set a 10-year deadline for embryos to be discarded. Therefore, the 10-year limit was 2009. We define before 2009 as phase 1 of the study and then proceeded based on the phase 1 research findings, after which phase 2 (from 2010 to 2014) was verified.

### Laboratory protocols

Laboratory protocols were performed as previously described [17–19]. The protocol for vitrification and warming was adapted Mukaida et al., as in our previous report [5]. This study was approved by the Ethics Committee of CGMH (CGMH2O1901329B0). Day 3 embryos were evaluated (66–68 h postinsemination/ICSI, respectively) and were classified using a modification of Veeck’s morphologic grading system [20].

All transferable embryos were assessed on day 3 for blastomere number and regularity as well as the presence and volume of cytoplasmic fragmentation. The embryos were graded on a scale of 0 to 4 according to a method modified as described elsewhere [21]; scores of 4 and 3 indicated eight cells with blastomeres of equal size, a score of 2 indicated eight cells with uneven blastomere sizes and no cytoplasmic fragments, a score of 1 indicated four or eight cells with > 20% fragmentation, and a score of 0 indicated pre-embryos with few blastomeres of any size with major or complete fragmentation. Embryos with a score of 4 were defined as top-quality embryos.

In our institute, patients may provide one or more frozen embryos for storage. Verbal and written consent includes informing the patient/couple that they will be contacted annually and that they are expected to make one of several choices regarding disposition of the embryos ((i) keep storing, (ii) transfer to another institute, (iii) discard, or (iv) donate for research). At the time of consent, patients acknowledge their understanding that they are expected to keep the KCGMH program informed of their current address and marital status (Taiwan Identity card spouse column) and to pay storage bills as long as they have embryos frozen at the facility. If the storage time is nearing expiration, the KCGMH makes a phone call at an appointed time. If the participants do not respond, second and third telephone calls are made 3 or 4 days after the initial call. If a couple does not respond after 3 calls, the couple is classified as a “lost contact.” If a couple decided to discontinue storage, they were asked to come back to the center to personally sign a document. A final telephone call is made approximately 6 months prior to the expiration date, as stipulated by the Taiwan National Artificial Reproduction Act.

### Statistical analysis

All data analyses were performed using SPSS for Windows (version 20.0). Categorical variables are expressed as percentages and continuous variables as medians (interquartile ranges [IQRs]) or means ± standard deviations (SDs), as appropriate. Visual inspection and the Shapiro-Wilk normality test were used to check for normality of distributions. A receiver operating characteristic (ROC) curve was used to determine the optimal cutoff of dosimetric data for embryo abandonment with a non-abandonment counterpart. After determination of the optimal cutoff using the ROC curve, a logistic regression model using categorical variables (endocrine parameters, patient’s age, age of the male partner, BMI, infertility diagnosis, ovarian stimulation protocol, duration of ovarian stimulation, maximal endometrial thickness, number mature oocytes retrieved, number of embryos transferred, transfer embryo score, use of ICSI, use of blastocyst-stage ET, number of frozen embryos, and pregnancy outcome) was used for confirmation of dosimetric significance. A full model, with the inclusion of all variables to adequately control for potential confounding, was implemented, and the results are presented as adjusted odds ratios (aORs) and 95% confidence intervals (95% CIs). An OR was considered statistically significant if its two-tailed *p* value was less than 0.05 or if its 95% CI did not include one.

## Results

### Phase 1

**Fig. 1:**
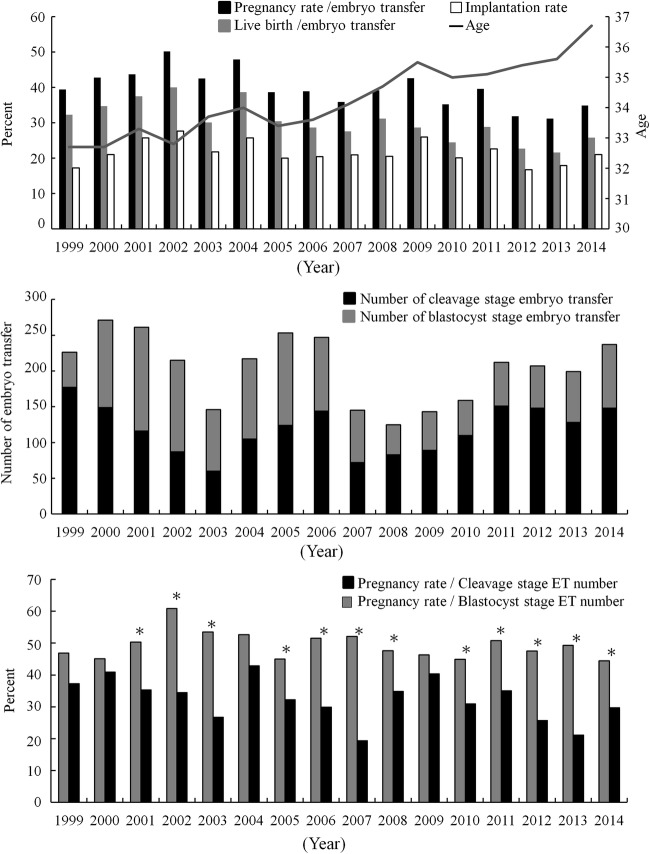
Annual IVF/ICSI outcome from 1999 to 2014 in KCGMH. **a** Annual pregnancy rate, live birth rate, implantation rate and patient age curve. **b** Numbers of annual blastocyst stage embryo transfers and cleavage-stage embryo transfers (**c**). Comparison of annual blastocyst stage embryo transfer and cleavage-stage embryo transfer pregnancy rates. *< 0.05

A total of 2414 IVF/ICSI cycles were performed between 1999 and 2009. General characteristics concerning the causes of infertility, assisted reproduction technique (IVF or ICSI), and outcome were noted and are presented in Table 1 and Fig. 1. The mean female age was 33.6 ± 4.6 years, and 15,602 oocytes were retrieved and then produced 12,308 embryos (including the cleavage and blastocyst stages) during these cycles. During this 10-year period, the annual pregnancy rate ranged from 31.2 to 50.2%, the live birth rate ranged from 22.7 to 40%, and the implantation rate (irrespective of age) ranged from 16.7 to 26% (Fig. 1a). With 5 years as a unit, the age of patients gradually increased, and the proportion of patients using BT decreased (Fig. 1a and b). The annual pregnancy rate from BT ranged from 44.4 to 50.8%, with statistically significant or higher rates than cleavage transfer in the same year (Fig. 1c).

**Table 1:** Demographic characteristics of 2414 Cycles that underwent IVF/ICSI from 1999 to 2009

IVF/ICSI cycles, *n*	2414
Infertility, *n*	
Primary	1528
Secondary	886
Duration of infertility years ± SD (range)	4.5 ± 3.2 (1–21)
Number of indications	
Tubal factor	516
Male	721
Endometriosis	369
Ovulatory factor	245
Unexplained and others	563
Mean female age, years ± SD (range)	33.6 ± 4.6 (19–49)
Number of cycles with at least one oocyte retrieved	2381
Number of cycles that underwent IVF	1647
Number of cycles that underwent ICSI	734
Number of cycles transferred	2218
Number of cycles of cleavage stage transfer	1044
Number of cycles of blastocyst stage transfer	1174
Endometrial thickness on day of hCG (mm)	1.2 ± 0.2
Estradiol (pg/mL) on hCG day	1662.3 ± 937.3
Progesterone (pg/mL) on hCG day	1.2 ± 0.3
Mature oocytes retrieved, *n*	15,602
No of mature oocytes retrieved	6.5 ± 4.2 (1–24)
Normal fertilization rate	78.8% (12,308/15,602)
Embryos produced, *n*	12,308
IVF/ICSI cycles with frozen embryos, *n* (%)	571 (23.6%)
Surplus frozen embryos, *n* (%)	1790 (14.5%)
Abandoned embryos, *n* (%)	965 (7.8%)

*n* number

A total of 1790 surplus embryos (14.5%) were frozen in 571 (23.5%) cycles (Table 1). A total of 53.9% (number = 965) of frozen embryos were abandoned from 1999 to 2009 (Table 2). Among the 335 couples who abandoned embryos, the mean female age was 31.6 ± 4.3 years. Female death and divorce were each the cause of one embryo disposal. The legal expiration time occurred in 265 couples, 234 of whom had lost contact for more than 5 years. A total of 68 couples signed documents authorizing embryo abandonment, with 30 choosing to discard the embryo and 38 choosing to donate the embryo for research. A total of 87.5% of couples abandoned 1 to 5 stored embryos, and 73.7% of couples with abandoned embryos received their first IVF treatment.

**Table 2:** Characteristics of abandoned IVF/ICSI embryo cycles from 1999 to 2009

Patients with abandoned embryo cycles, *n*	335
Abandoned embryos, *n*	965
Mean female age, years ± SD (range)	31.6 ± 4.3 (20–42)
Primary/secondary infertility, *n*/*n*	203/132
Infertility duration, years ± SD	4.0 ± 1.1
Patients with first time IVF, *n* (%)	247(73.7%)
Pregnancy rate per transfer cycle (%)	74.4%
Patients abandoning embryo due to legal expiration, *n*	265
Lost contact	239
Lost contact more than 5 years	234
With contact but without completed documentation	26
Patients abandoning embryos with documentation, *n*	68
Chose to discard	30
Chose donation for research	38
Divorce or death, *n*	2
Patients who stored different numbers of embryos, *n*	
1 to 5 embryos	293 (87.5%)
6 to 10 embryos	35 (10.4%)
> 11 embryos	7 (2.1%)

We performed logistic regression analysis to determine the relationships of multiple variables with embryo abandonment or non-abandonment from 1999 to 2009 (Table 3). The crude ORs indicated that retrieval of ≥ 6 mature oocytes, positive pregnancy outcome, production of ≥ 6 embryos, young female age ≤ 35, freezing of ≥ 2 embryos, using blastocyst transfer, and transfer of more high-quality embryos were associated with embryo abandonment. After adjustment for confounding, the only factors that remained significant were positive pregnancy results (aOR = 4.38, 95% CI = 3.17 to 6.04), number ≥ 2 of frozen embryos (aOR 3.68, 95% CI 3.10, 4.38), ≥ 6 embryos produced (aOR 1.68, 95% CI 1.03, 2.73), and using blastocyst transfer (aOR 2.46, 95% CI 1.64, 3.69). A total of 88.3% (number = 234) of couples who lost contact for more than 5 years had their frozen embryos discarded because of legal time expiration.

**Table 3:** Conditional logistic regression of the association of embryo abandonment with different variables from 1999 to 2009

Variable	Crude OR	95% CI	*p* value	Adjusted OR	95% CI	*p* value
Number of mature oocytes (≥ 6 vs < 6)	6.40	[4.96–8.22]	< 0.001	0.91	[0.57–1.46]	*NS*
Embryos produced (≥ 6 vs < 6)	8.46	[6.53–10.95]	< 0.001	1.68	[1.03–2.73]	< 0.035
No. of embryos transferred (≥ 2 vs < 2)	1.32	[1.05–1.12]	0.019	0.54	[0.25–1.18]	*NS*
Score of embryos transfer (> 8 vs ≤ 8)	1.90	[1.51–2.40]	< 0.001	1.95	[0.91–4.21]	*NS*
Blastocyst transfer vs cleavage transfer	7.29	[5.31–10.02]	< 0.001	2.46	[1.64–3.69]	< 0.001
No. of frozen embryos (≥ 2 vs < 2)	5.51	[4.48–5.93]	< 0.001	3.68	[3.10–4.38]	< 0.001
Pregnancy (yes vs no)	5.33	[4.12–6.88]	< 0.001	4.38	[3.17–6.04]	< 0.001
Female age (≤ 34 vs > 34)	2.67	[2.05–3.48]	< 0.001	1.37	[0.97–1.93]	*NS*

*NS* not significant

### Phase 2

A total of 1753 IVF/ICSI cycles were performed from 2010 to 2014 (Table 4). We also analyzed factors associated with embryo abandonment or the non-abandonment counterpart during this period (Table 5). There were 20 couples with abandoned embryos and 129 couples with frozen embryos who lost contact for more than 5 years. We defined the 129 couples who lost contact for more than 5 years as the possible embryo abandonment group according phase 1 findings. In addition, 118 patients (91.5%) stored 1 to 5 stored embryos (Table 5).

**Table 4:** Demographic characteristics of 1753 cycles that underwent IVF/ICSI from 2010 to 2014

IVF/ICSI cycles, *n*	1753
Infertility, *n*	
Primary	1140
Secondary	613
Duration of infertility years ± SD (range)	4.2 ± 2.8 (1–20)
Number of indications	
Tubal factor	402
Male	353
Endometriosis	201
Ovulatory factor	293
Unexplained and others	504
Mean female age, years ± SD (range)	35.8 ± 4.4 (18–48)
Number of cycles with at least one oocyte retrieved	1693
Number of cycles that underwent IVF	1082
Number of cycles that underwent ICSI	611
Number of cycles fresh transferred	1327
Number of cycles of cleavage stage transfer	712
Number of cycles of blastocyst stage transfer	615
Endometrial thickness on day of hCG (mm)	1.3 ± 0.2
Estradiol (pg/mL) on hCG day	2232.7 ± 1874.7
Progesterone (pg/mL) on hCG day	1.0 ± 0.1
Mature Oocytes retrieved, *n*	11,129
No of mature oocytes retrieved	6.4 ± 4.1 (1–32)
Normal fertilization rate	69.7% (7756/11129)
Embryos produced, *n*	7756
IVF/ICSI cycles with frozen embryos, *n* (%)	690 (39.3%)
Surplus frozen embryos, *n* (%)	2767(35.6%)

*n* number

**Table 5:** Characteristics of abandoned and possibly abandoned embryo cycles from 2010 to 2014

Patients with abandoned embryo cycles, *n*	20
Patients with frozen embryo but no contact for more than 5 years, *n*	129
Mean female age, years ± SD (range)	32.9 ± 3.8 (22–43)
Primary/secondary infertility, *n*/*n*	84/65
Infertility duration, years ± SD	4.1 ± 1.3
Patients with first time IVF, *n* (%)	108(72.5%)
Pregnancy rate per transfer cycle (%)	66.9%
Patients abandoning embryos with documentation, *n*	20
Chose to discard	18
Chose donation for research	2
Divorce or death, *n*	0
1 to 5 embryos	118
6 to 10 embryos	27
> 11 embryos	4

*n* number

Logistic regression analysis of the relationship of treatment characteristics with embryo abandonment and possible abandonment or the non-abandonment counterpart from 2010 to 2014 (Table 6) indicated that retrieval of ≥ 6 oocytes, positive pregnancy outcome, production of ≥ 6 embryos, young female age, freezing of number ≥ 2 embryos, and using blastocyst transfer were associated with embryo abandonment and possible abandonment. After adjustment for confounding factors, positive pregnancy outcome (aOR = 2.59, 95% CI = 1.73 to 3.89), young female age ≤ 35 (aOR = 1.56, 95% CI = 1.06 to 2.39), freezing ≥ 2 embryos (aOR = 4.73, 95% CI = 2.70 to 8.27), and transfer of more high-quality embryos (aOR = 2.10, 95% CI = 1.26 to 3.49) remained associated with embryo abandonment and possible abandonment.

**Table 6:** Conditional logistic regression of the association of embryo abandonment or possible abandonment with different variables from 2010 to 2014

Variable	Crude OR	95% CI	*p* value	Adjusted OR	95% CI	*p* value
Number of mature oocytes (≥ 6 vs < 6)	8.61	[5.27–14.05]	< 0.001	1.36	[0.68–2.69]	*NS*
Embryos produced (≥ 5 vs < 5)	11.73	[7.01–19.62]	< 0.001	1.73	[0.82–3.64]	*NS*
No. of embryos transferred (≥ 2 vs < 2)	2.35	[1.59–3.47]	< 0.001	0.89	[0.47–1.54]	*NS*
Score of embryos transfer (> 8 vs ≤ 8)	4.10	[2.91–5.78]	< 0.001	2.10	[1.26–3.49]	0.04
Blastocyst transfer vs cleavage transfer	1.92	[1.58–2.34]	< 0.001	1.03	[0.82–1.32]	*NS*
No. of frozen embryos (≥ 2 vs < 2)	12.957	[8.43–19.91]	< 0.001	5.77	[3.40–9.27]	< 0.001
Pregnancy (yes vs no)	4.73	[3.37–6.65]	< 0.001	2.59	[1.73–3.89]	< 0.001
Female age (≤ 35 vs > 35)	3.67	[2.54–5.30]	< 0.00	1.56	[1.06–2.39]	0.025

*NS* not significant

Data for phase 1 and phase 2 were then combined (from 1999 to 2014). After determination of the optimal cutoff using an ROC curve, the relationships of treatment characteristics with embryo abandonment and possible abandonment or the non-abandonment counterpart again were subjected to logistic regression analysis. After adjustment for confounding factors, positive pregnancy outcome (aOR = 3.37, 95% CI = 2.63 to 4.33), young female age ≤ 35 (aOR = 1.50, 95% CI = 1.14 to 1.97), ≥ 6 embryos produced (aOR 1.57, 95% CI 1.04, 2.37), production of ≥ 2 frozen embryos (aOR = 7.58, 95% CI = 5.79 to 9.92), using blastocyst transfer (aOR 1.26, 95% CI 1.10, 1.45), and transfer of more high-quality embryos (aOR = 2.43, 95% CI = 1.64 to 3.61) remained associated with embryo abandonment and possible abandonment (Table 7).

**Table 7:** Conditional logistic regression of the association of embryo abandonment or possible abandonment with different variables from 1999 to 2014

Variable	Crude OR	95% CI	*p* value	Adjusted OR	95% CI	*p* value
Number of mature oocytes (≥ 7 vs < 7)	8.01	[6.30–10.19]	< 0.001	1.31	[0.88–1.95]	*NS*
Embryos produced (≥ 6 vs < 6)	9.99	[7.85–12.74]	< 0.001	1.57	[1.04–2.37]	0.031
No. of embryos transferred (≥ 2 vs < 2)	3.04	[2.26–4.08]	< 0.001	0.64	[0.39–1.07]	*NS*
Score of embryos transfer (> 8 vs ≤ 8)	4.91	[3.87–6.22]	< 0.001	2.43	[1.64–3.61]	< 0.001
Blastocyst transfer vs cleavage transfer	2.29	[2.04–2.57]	< 0.001	1.26	[1.10–1.45]	0.001
No. of frozen embryos (> 2 vs ≤ 2)	15.40	[12.32–19.23]	< 0.001	7.58	[5.79–9.92]	< 0.001
Pregnancy (yes vs no)	5.31	[4.33–6.50]	< 0.001	3.37	[2.63–4.33]	< 0.001
Female age (≤ 35 vs > 35)	3.37	[2.67–4.25]	< 0.001	1.50	[1.14–1.97]	0.004

*NS* not significant.

## Discussion

The key result of the present study of 2414 IVF/ICSI cycles in which there was embryo abandonment under the National Assisted Reproduction Act of Taiwan indicated that patients age ≤ 35 with blastocyst transfer, a positive pregnancy outcome with ≥ 6 embryos produced, and ≥ 2 frozen embryos were the major factors associated with the discarding of frozen embryos from 1999 to 2009. Under legal time expiration, elective BT and a freezing strategy for surplus embryos resulted in a 92.2% embryo utilization rate. However, 53.9% of frozen embryos were abandoned from 1999 to 2009.

Can any clinical treatment or laboratory indicator be used to identify couples who choose to discard embryos? The presence of such indicators seems plausible, and different therapists may speculate about such indicators based on their observational experiences, but there is no such evidence in the literature. Previous studies of abandoned embryos have focused on ethical, legal, and patient decision issues, rather than IVF strategies. Our study examined the factors associated with patient abandonment of embryos produced from elective blastocyst culture. A study from 2001 found that a positive outcome of the original IVF treatment and a short maximum legal time of cryopreservation were the most common reasons why couples discarded their embryos [22]. Compared to our study, this previous study reported a similar percentage of couples who had frozen embryos (24.4%), but a higher percentage of couples who did not use their embryos (29.8%).

A study in China also examined factors associated with the disposition of frozen embryos after a live birth following IVF. The results indicated that the preference for embryo disposal was associated with the number of children, storage duration, and the couple’s education [23]. In contrast, Apte et al. [24] reported that patient age and outcome of the first cycle did not significantly influence patient preference for embryo disposal. However, 70% of interviewed patients reported feeling distress when faced with this decision because this study had a duration of only 1 year [24].

Nachtigall et al. [25] suggested that patient decisions about the disposition of frozen embryos would be facilitated by clear presentations of procedures, counseling, and guidance about the available options. Nevertheless, in spite of the clear informed consent procedure used in our clinic, many patients required multiple communications to obtain responses. Thus, clinics should anticipate that many patients will not return to claim stored embryos, others will not provide a final directive regarding embryo disposal, and others may change their views over time [7]. Previous studies that examined patient perspectives reported that couples face difficulties when deciding whether or not to continue storing their frozen embryo(s). The factors affecting this decision include embryo conceptualization, information, and support provided by the medical institution, quality of the embryo(s), and life events [12, 15, 25–31]. Of course, a previous poor pregnancy experience from embryo transfer, such as abortion, ectopic pregnancy, or complications from treatment-derived ovarian hyperstimulation, will also affect the patient’s desire to abandon frozen embryos. However, our study indicated that these factors did not have a significant effect when deciding to abandon an embryo.

Decisions about the disposition of abandoned embryos are particularly difficult because of the sensitive nature of this issue regarding the moral status of human embryos, but there are also legal concerns—what if the clinic simply forgot to contact someone who in fact wants their embryos? This can cause clinics to hesitate when deciding to discard an abandoned embryo.

A substantial number of our couples (79.1% = 265/335) (Table 2) did not return for their cryopreserved embryos, particularly if the previous IVF treatment led to a successful pregnancy. According to our study, the guidelines of the National Assisted Reproduction Act, which sets an embryo storage time of 10 years, seem suitable, although most patients who have lost contact at 5 years ultimately decide to discard the embryo(s). It seemed to match the study by Bruno et al., in which empirical research showed that nearly 70% of the patients delayed the decision for 5 years or more [28]. Therefore, it can be assumed that after 5 years without contact with couples or individuals, the embryo is considered possibly abandoned [32]. The establishment of a legal time limit makes it clear to patients, fertility clinics, and storage facilities what will be done with embryos in storage, irrespective of their status as abandoned once the time limit has lapsed [9].

Our results indicated that a successful pregnancy outcome after embryo transfer was the major factor associated with embryo abandonment. Younger women (age ≤ 35 years) with a good prognosis for successful pregnancy were also more likely to abandon their embryos. However, the basic problem of how to avoid the production of excessive embryos may be the most important issue. It is only to pursue the pregnancy rate of treatment to meet the patient’s expectation of maintaining proper health and achieving a live birth and to balance the production of embryos. Taiwan society has different ethical perceptions of embryos and gametes. Moreover, it is not permitted to donate surplus embryos to another couple, but oocyte donation is permitted under the Taiwan Reproduction Act. However, oocyte cryopreservation is no longer experimental, and one of its rapidly growing indications is elective fertility preservation. Frozen and fresh oocytes produce similar IVF outcomes, and freezing is not associated with additional obstetrical or perinatal morbidities [33, 34]. However, there is no sufficient evidence to support its practice; therefore, its use in IVF remains uncertain. It can be hypothesized that oocyte cryopreservation would decrease the possibility of abandoned embryos. How to design a suitable and cost-effective embryo production algorithm and related issues is also worthy of exploration.

Our study had some limitations. Some patient information that might have been helpful, such as religious and personal beliefs regarding the moral status of the embryo, was not available. Use of time-lapse culture with morphokinetic embryo selection or preimplantation genetic testing for aneuploidy prior to freezing was limited during the study period. Thus, we cannot provide a definitive answer about the relationship of intensive embryo selection procedures with the decision to discard an embryo.

In conclusion, embryo abandonment was strongly associated with pregnancy after IVF treatment and with the production of a large surplus of frozen embryos. For elective blastocyst stage transfer according to the Taiwan National Assisted Reproduction Act, a young female age ≤ 35 with a positive pregnancy status, the production of ≥ 6 embryos, and the cryopreservation of ≥ 2 blastocysts may be more likely to be associated with the abandonment of embryos in the future.
